# Study Protocol of a Prospective, Monocentric, Single-Arm Study Investigating the Safety and Efficacy of Local Ablation of Symptomatic Uterine Fibroids with US-Guided High-Intensity Focused Ultrasound (HIFU)

**DOI:** 10.3390/jcm12185926

**Published:** 2023-09-12

**Authors:** Dieter M. Matlac, Tolga Tonguc, Nikola Mutschler, Florian Recker, Olga Ramig, Holger M. Strunk, Tatjana Dell, Claus C. Pieper, Martin Coenen, Christine Fuhrmann, Oregan Vautey, Eva-Katharina Egger, Jim Küppers, Rupert Conrad, Markus Essler, Alexander Mustea, Milka Marinova

**Affiliations:** 1 Department of Gynecology and Gynecological Oncology, University Hospital Bonn, University Bonn, 53127 Bonn, Germany; 2 Department of Diagnostic and Interventional Radiology, University Hospital Bonn, University Bonn, 53127 Bonn, Germany; 3 Department of Neuroradiology, University Hospital Bonn, University Bonn, 53127 Bonn, Germany; 4 Department of Obstetrics and Prenatal Medicine, University Hospital Bonn, University Bonn, 53127 Bonn, Germany; 5 Medical Center, University Bonn, 53115 Bonn, Germany; 6 Clinical Study Core Unit Bonn, Institute of Clinical Chemistry and Clinical Pharmacology, University Hospital Bonn, University Bonn, 53127 Bonn, Germanychristine.fuhrmann@ukbonn.de (C.F.); 7 Department of Nuclear Medicine, University Hospital Bonn, 53127 Bonn, Germany; 8 Department of Psychosomatic Medicine and Psychotherapy, University Hospital Muenster, 48149 Muenster, Germany

**Keywords:** symptomatic uterine fibroids, high-intensity focused ultrasound, structural integrity of uterine tissue, US and MRI elastographic methods

## Abstract

Uterine fibroids are the most common benign tumors of the uterus. Approximately 20–50% of women with myomas experience a variety of symptoms such as vaginal bleeding, abdominal pain, pelvic pain and pressure, and urological problems, possibly interfering with fertility and pregnancy. Although surgery remains the standard treatment option for fibroids, non-invasive therapeutic options, such as high-intensity focused ultrasound (HIFU), have emerged over the last dec ade. During HIFU, ultrasound is focused on the target tissue causing coagulation necrosis. HIFU has, meanwhile, become an established method for treating uterine fibroids in many countries. Clinical data have shown that it effectively alleviates fibroid-related symptoms and reduces fibroid size with a very low rate of side effects. However, there is a lack of data on how this treatment affects laboratory parameters and structural features of uterine tissue. As our center is the only one in German-speaking countries where ultrasound-guided HIFU technology is currently established, the aim of this prospective, monocentric, single-arm trial is not only to evaluate the safety and efficacy of local US-guided HIFU in symptomatic uterine fibroid patients according to GCP standards but also to explore its effects on blood parameters and the structural integrity of uterine tissue using elastographic methods.

## 1. Uterine Fibroids

Uterine fibroids are the most common benign tumors of the uterus. As their growth depends on the female sexual hormone estrogen, fibroids mostly occur in women of childbearing age. In a methodologically sound systematic review, the following twelve risk or protective factors were identified: black race, age, premenopausal state, hypertension, family history, time since last birth, and food additive and soybean milk consumption increase fibroid risk; the use of oral contraceptives or the injectable contraceptive depot medroxyprogesterone acetate and smoking in women with low body mass index and parity are protective factors [1]. Other large datasets suggest that a clinical history of obesity, current alcohol use, and chronic psychological stress may increase the risk/prevalence of uterine fibroids [2,3]. Interestingly, an increase in the frequency of fibroids is shown in women with a history of benign breast disease and particularly of breast biopsies [4]. Moreover, the evolution of uterine fibroids during pregnancy and puerperium appears to follow a non-linear trend, with systematic enlargement observed in the first trimester, while changes during the second and third trimesters are supported by inconsistent evidence, and the overall modifications of myomas during this period remain uncertain [5].

Most uterine fibroids do not cause any symptoms, only 25–30% of the patients experience fibroid-associated symptoms [6]. These include abnormal uterine bleeding, pain or pressure in the lower abdomen with potential effects on the bladder or rectum, and even dyspareunia and fertility [7]. Especially submucosal myomas (classified as type 3 according to the FIGO classification) are notably associated with reduced implantation rates, cumulative pregnancy rates, and live birth rates [8]. Moreover, their negative influence on IVF outcomes becomes more pronounced with larger size and an increased number of such myomas. In addition, uterine fibroids can be associated with pregnancy complications [9]. 

As long as fibroids do not cause any symptoms and there is no evidence of malignancy, no treatment is necessary. For symptomatic fibroids, there are various treatment options, including pharmacological (hormone) therapy, surgical therapies such as myomectomy or hysterectomy, and minimally invasive therapies such as uterine artery embolization (UAE) [7] and local ablation with high-intensity focused ultrasound (HIFU) as a non-invasive procedure [10]. 

## 2. Pharmacological Therapy of Uterine Myomas

Several groups of pharmacological agents, such as gonadotropin-releasing hormone agonists, oral combined contraceptives, progesterone receptor modulators, and intrauterine progestogen coils, are available for drug therapy of symptomatic uterine fibroids [11,12]. The progesterone receptor modulator ulipristal acetate (Esmya^®^) was commonly used in the past for symptom relief and fibroid volume reduction prior to pending surgery. However, the usefulness of this drug has been out of perspective since 2018 due to observed side effects and an unfavorable risk–benefit profile [13]. 

The most recent change in the pharmacological therapy of fibroids has been the approval of Ryeqo^®^ (Gedeon Richter) in June 2021. Ryeqo^®^ is a combinational drug containing relugolix, a selective GnRH-receptor antagonist, estradiol, and norethisterone acetate. It has been shown to significantly reduce fibroid-associated symptoms in two approval studies. The combination of these three drugs is supposed to reduce fibroid-induced symptoms while keeping symptoms due to estrogen shortage low and preventing a higher risk of endometrial carcinoma [14].

## 3. Gynecological Surgical Treatment of Uterine Fibroids

Surgical therapy remains the most direct treatment for uterine fibroids as a primary therapeutic option. It can either be performed laparoscopically or through an abdominal incision. Depending on the size, number, location of the fibroids, and the patient’s desire for uterus preservation, there are different surgical approaches. For submucosal fibroids, hysteroscopy can be usually performed. For subserosal fibroids, enucleation by laparoscopy may be an option, and if laparoscopy is not feasible, a surgical procedure by laparotomy is necessary. Removal of the uterus is indeed the most effective treatment, although several factors have to be considered. If removal of the uterus is desired, a choice can be made between open and laparoscopic procedures depending on the size of the uterus. Moreover, in rare cases (under 0.03%), malignant sarcoma may be present [15]. Considering aspects of oncological safety, morcellement of the uterus is avoided in our clinic.

## 4. HIFU for Uterine Fibroids

High-intensity focused ultrasound (HIFU; high-intensity focused ultrasound) represents an innovative and non-invasive therapeutic option for symptomatic uterine fibroids [16]. This technique allows targeted thermal ablation of solid tumors accessible to sonography [17]. The decision on the indication for HIFU treatment is made individually for each patient in an interdisciplinary team of the HIFU center at the University Hospital Bonn. Fibroid ablation with HIFU represents a low-risk intervention compared with other procedures with few and, in the rarest cases, severe side effects [18].

In contrast to diagnostic ultrasound, HIFU generates much higher energies in the target area during ablation. The ultrasound waves are focused by special transducers in the lesion, and the focus measures only a few millimeters. This results in local heating of the target tissue up to more than 60 °C, inducing coagulation necrosis. 

In recent years, HIFU has been increasingly used for the local therapy of different benign and malignant solid tumors [19]. The clinical value of HIFU treatment for uterine fibroids has been investigated in numerous studies involving large patient populations over the past decade. All of these studies have described the procedure as safe and effective, with a significant clinical benefit for patients through the reduction in myoma-related symptoms. Symptom reduction and a decrease in fibroid volume have been shown in all types of treated fibroids [20]. Special attention should be given to submucosal fibroids, as they may have potential negative effects on the fertility of patients in their childbearing age [8]. Therefore, a comprehensive and individual evaluation of the prospects and feasibility of HIFU treatment is warranted in this context.

Side effects are rare and less frequent than after surgical procedures like myomectomy and hysterectomy [16]. Moderate pain during the procedure, which is performed under conscious sedation, may last for a few hours in some cases, and cutaneous/subcutaneous edema of the anterior lower abdominal wall in the acoustic pathway are common side effects [21]. However, fever, urinary tract infections, hematuria, bowel lesions, and back pain are possible, but these are very rare nowadays.

The duration of the procedure depends on the size, number, and location of the target fibroids and lasts normally 1–2 h but may take up to 3–4 h. The HIFU treatment can be performed on an outpatient basis; after a rest period of four to six hours, the patient can usually leave the clinic and resume her normal daily routine the next day. HIFU treatment of fibroids requires good coordination between interventionalists, anesthesiologists, and gynecologists, as previously described [22]. In the course of this prospective study, the patients are hospitalized for one night in the local gynecology department in order to accurately evaluate the treatment-associated side effects. 

## 5. Rationale of the Study

The study has a prospective, monocentric, and single-arm design and is intended to evaluate the safety and efficacy of local therapy with ultrasound-guided HIFU in patients with symptomatic uterine fibroids according to GCP standards. To the best of our knowledge, the University Hospital Bonn is currently the only center in German-speaking countries where an ultrasound-guided HIFU system (TTS, tumor therapeutic system, Chongqing Haifu Medical Technology, China) is available and in use as opposed to several other centers that use MRI-guided HIFU. The evaluation of study results is based on clinical gynecological investigation, records, and assessment of side effects; the UFS-QOL questionnaire on disease-related symptoms (symptom severity score) and health-related quality of life (QOL); and changes in fibroid volume over time using MRI [19]. This present study aims to identify patients who would benefit the most from HIFU treatment and collect data to plan a confirmatory phase III trial assessing the efficacy of combining HIFU with other therapies. Additionally, this study investigates the effects of HIFU treatment on laboratory parameters, including its potential to induce an immune response. Another exploratory endpoint is an assessment of elastographic features of uterine fibroid tissue before and after HIFU treatment, utilizing both sonographic share wave elastography and MR imaging techniques.

## 6. Trial Design

**Figure 1:**
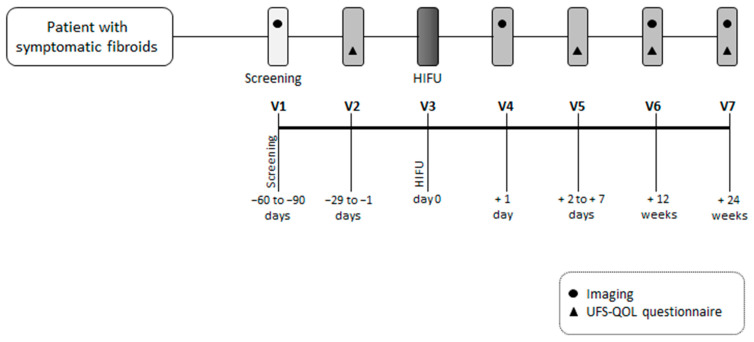
Intervention scheme.

**Figure 2:**
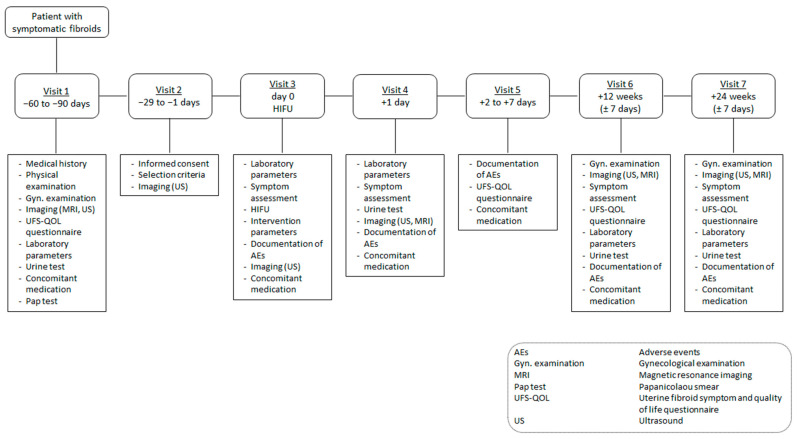
Trial flow.

The current investigation is a prospective, monocentric, single-arm, and open-label study (Figure 1 and Figure 2). The entire study duration per patient is 8 months, including screening visits. The HIFU procedure typically lasts for 2–5 h, with a hospital stay for one night. Each patient is monitored for a follow-up period of 6 months. The clinical trial will be conducted in the interdisciplinary HIFU center at the University Hospital Bonn, and all investigators meet the requirements to perform the planned study-specific examinations and therapies.

## 7. Study Population

Twenty eligible patients will be recruited for this study. Potential participants who present at the gynecology department will be informed about the study and given adequate time to reflect before providing written informed consent to participate. Patients who do not meet the inclusion criteria (outlined in Table 1) will not be included in the study.

Fertile study patients will be informed that pregnancy or breastfeeding are contraindications to participating in the study and will be instructed to use reliable contraceptive methods throughout the study period. Reliable contraceptive methods with a Pearl Index below 1 include hormonal methods, barrier methods, and hormonal medication. Patients will be required to abstain from sexual intercourse during the first month after the HIFU procedure, and transvaginal ultrasound should be avoided during this period. Eligible patients will be mainly recruited from the outpatient clinic of the Department of Gyneco-logy, but patients referred by external gynecologists or those who present themselves will be also included.

The interdisciplinary uterine fibroid conference, which convenes weekly, will determine whether HIFU treatment is indicated for each patient on an individual basis. Potentially suitable patients will receive detailed information about their participation in the study and the procedures involved by the investigator. The study has been initiated after professional ethical consultation by the responsible ethics committee at the University Hospital Bonn.

## 8. Measurements

### 8.1. Primary Endpoints

The primary objective of this study is to evaluate the safety and tolerability of local HIFU treatment in patients with symptomatic uterine fibroids. This involves evaluating incidents and product defects as well as adverse events after HIFU therapy. Adverse events will be graded according to the Common Terminology Criteria for Adverse Events (CTCAE, Version 5.0), with grades 1–5 indicating increasing severity.

In addition to the frequency and severity of adverse events according to CTCAE, the expected treatment-associated adverse events will be recorded, including pain during and immediately after the HIFU procedure (measured on a pain scale NRS/VAS 0–10, with 0 indicating no pain and 10 indicating the most severe pain), pain within the first day after the procedure, skin edema, skin redness, skin burning, ascites, cystitis, vaginal discharge, and vaginal bleeding.

### 8.2. Secondary Endpoints

The following objectives are defined as secondary endpoints:

- Evaluation of symptom severity score (SSS) and health-related quality of life score using UFS-QOL questionnaire (baseline scores versus scores 1 week, 3 months, and 6 months after HIFU procedure).
- Evaluation of fibroid volume using MRI measurements (baseline volume versus volume 1 week, 3 months, and 6 months after HIFU procedure).
- Evaluation of the correlation between intervention parameters (sonication time, treatment time, total energy, and energy per milliliter of fibroid volume), achieved non-perfused volume, fibroid type according to Funaki classification, and fibroid shrinkage over time.
- Evaluation of tissue stiffness properties of uterine fibroids using elastography measurements on transabdominal ultrasound and MRI before and after HIFU procedure.
- Effects of HIFU treatment on blood parameters.

## 9. Data Collection

Several kinds of data will be assessed in this study at each visit (Table 2). Data related to symptoms will be gained through communication with the patients and objectivation via established questionnaires. In addition, blood samples will be taken and analyzed for several parameters with emphasis on immune system-related parameters. Moreover, changes in the physical quality of fibroid tissue before and after HIFU treatment will be monitored through follow-up ultrasound and MRI examinations, including elastography.

## 10. Statistical Analysis

Data from this prospective observational longitudinal study will be analyzed using Stata (latest version, StataCorp, StataCorp LP, College Station, TX, USA) and SPSS (latest version, SPSS Inc., Chicago, IL, USA). Evaluation of changes in fibroid volumes, symptom severity, and quality of life scores will be performed by using a longitudinal mixed model considering values at baseline and each follow-up as dependent variables. The influence of various interventional parameters (total energy, sonication time, and power) and assignment to corresponding Funaki types on non-perfused volumes and volume reduction over time will be analyzed using logistic regression and a mixed model, respectively. A *p*-value of < 0.05 is considered statistically significant.

## 11. Strengths and Limitations

The present study offers several strengths, including its prospective design and focus on ultrasound-guided HIFU therapy in a German cohort. Notably, the University Hospital Bonn is currently the only center in Germany-speaking countries where US-guided HIFU technology is available and in use. For this reason, the primary endpoint of the study is to investigate the incidence and severity of treatment-related adverse events using the CTCAE classification. In addition to evaluating patients’ symptoms and well-being using standardized questionnaires, this study also employs innovative diagnostic modalities such as sonographic and MRI-based technologies to assess fibroid stiffness and provide a more comprehensive understanding of the effects of HIFU treatment beyond just changes in fibroid size. Moreover, the study focuses on early ablation-related laboratory changes and potential inflammatory reactions. While the strength of the study lies in its prospective design and the use of ultrasound-guided HIFU therapy on a German cohort, the limitation is referred to the relatively small sample size and the inability to directly compare the results to other treatment methods due to the single-arm study design.

## 12. Summary

Previous clinical research has demonstrated that high-intensity focused ultrasound (HIFU) is a non-invasive and low-risk treatment option for symptomatic uterine fibroids, as long as the fibroids are accessible to therapeutic ultrasound. HIFU provides effective symptom control while avoiding the potential risks and drawbacks of surgery, which is currently the standard treatment option. This study assesses the safety, efficacy, and clinical outcomes of US-guided HIFU treatment in 20 patients with symptomatic fibroids. The study aims to identify patients who would benefit most from this innovative treatment compared to other methods and to collect data that will serve as a foundation for future research on diagnostic and therapeutic approaches.

## Figures and Tables

**Table 1:** Selection criteria for HIFU treatment and study participation.

Inclusion Criteria	Exclusion Criteria
− Age ≥ 18 years − Written informed consent − Ability to follow study instructions and participate in required study visits − Fibroid visualization on diagnostic ultrasound − Minimum fibroid diameter of 2 cm − Maximum fibroid diameter ≤ 12 cm − No evidence of malignancy − Safe acoustic access path to the fibroid − Distance between skin surface and deepest fibroid regions of max. 11 cm − Fibroid-related symptoms − Patient’s MRI suitability	− Current pregnancy or breastfeeding − Suspected malignancy − Abnormal cervical cancer screening result − Acute cystitis − Acute infection (e.g., pneumonia) − Pedunculated or subserosal fibroids − Thick scar tissue on the skin or in the acoustic pathway − History of ileum conduit − Simultaneous participation in a clinical trial up to 30 days prior to participation in this clinical trial − Non-eligibility for conscious sedation

**Table 2:** Schedule of activities at each visit.

	Visit 1 −60 to −90 Days Screening	Visit 2 −29 to −1 Days Baseline	Visit 3 Day 0	Visit 4 +1 Day	Visit 5 +2 to +7 Days	Visit 6 +12 Weeks (±7 Days)	Visit 7 +24 Weeks (± 7 Days)
Selection criteria	(X)	X					
Informed consent	(X)	X					
Medical history	X						
Physical examination	X						
Vital signs	X						
Gynecological examination	X					X	X
Pap test	X						
Laboratory parameters	X	X	X	X		X	X
Urine test	X			X		X	X
MRI	X			X		X	X
Ultrasound inc. elastography	(X)	X	X	X		X	X
Records of concomitant medications	X		X	X	X	X	X
Records of pre-treatments	X						
Bowel preparation		X (day −1)					
Premedication		X					
Urinary catheter			X				
Preparation of abdominal wall			X				
HIFU treatment			X				
Assessment of pain (VAS 0–10)			X	X	X	X	X
Assessment of treatment-related AEs and SAEs			X	X	X	X	X
Assessment of additional AEs and SAEs			X	X	X	X	X
UFS-QOL		X			X	X	X
Imaging assessment		X	(X)	X		X	X
Intervention parameters (J/mL)			X				
NPV (%)				X			

AE: Adverse event; Gyn. Examination: Gynecological examination; HIFU: High-intensity focused ultrasound; MRI: Magnetic resonance imaging; NPV: Non-perfused volume; Pap test: Papanicolaou test, Papanicolaou smear; SAE: Serious adverse event; UFS-QOL: Uterine Fibroid Symptom and Quality of Life questionnaire; US: Ultrasound; VAS: Visual analog scale.

## Data Availability

This article represents a study protocol. Data will be generated in the course of the study and published later.
